# A rare transcript homozygous variants in CLRN1(USH3A) causes Usher syndrome type 3 in a Chinese family

**DOI:** 10.1186/s13023-024-03348-x

**Published:** 2024-09-20

**Authors:** Suyang Wang, Chen Yang Xu, Yiming Zhu, Wenjuan Ding, Jieyu Hu, Baicheng Xu, Yufen Guo, Xiaowen Liu

**Affiliations:** 1 Department of Otolaryngology-Head and Neck Surgery, Lanzhou University Second Hospital, No. 82 Cuiyingmen, Lanzhou, Gansu 730030 PR China; 2 Department of Otolaryngology-Head and Neck Surgery, Maternal and Child Health Hospital of Gansu Province, Lanzhou, China; 3 Department of Otolaryngology-Head and Neck Surgery, Gansu Provincial Hospital, Lanzhou, Gansu Province China

**Keywords:** Usher syndrome type 3, *CLRN1*, Isoform, Plasmid

## Abstract

**Background:**

Usher syndrome type 3 (USH3) is an autosomal recessive inherited disorder caused by pathogenic variants in the CLRN1 gene.

**Object:**

To evaluate the genotype-phenotype correlation of Usher syndrome type 3 (USH3) in a deaf-blind Chinese family of 3 generations with 2 patients.

**Methods:**

We collected blood samples and clinical data from all of the pedigree family members. Genomic DNA was isolated from peripheral leukocytes using standard method. Targeted next generation sequencing and Sanger sequencing were performed to find the pathogenic variants in this family. Digital PCR and plasmid overexpression assay were used to verify the pathogenicity of variant sites in different transcripts.

**Results:**

All patients developed bilateral sensorineural hearing loss (SHL), progressive vision loss and nyctalopia. NGS of genes for Usher syndrome, deafness and retinal dystrophy identified a locus mutation in *CLRN1* that caused completely different amino acid changes in different transcripts[*CLRN1*:c.474T > A(P.Cys158Ter) at NM_001256819.2 or c.302T > A(p.Val101Asp) at NM_174878.3], and plasmid overexpression experiments confirmed that the c.474T > A(P.Cys158Ter, NM_001256819.2) was a pathogenic variant which has never been associated with Usher syndrome in China, and the transcript of this mutation was not the version commonly found worldwide.

**Conclusions:**

The *CLRN1*c.474T > A(NM_001256819.2) mutation is the causative variant in the Chinese family with USH3. The pathogenicity of different transcripts should be particularly considered in pathogenicity analysis.

## Introduction

Usher syndrome(USH) is a serious genetic disorder responsible for the combined loss of hearing and vision [1].USH was first described by Scottish ophthalmologist Charles Usher. It is estimated that the incidence of USH in patients with congenital deafness is approximately 3.5/100,000 to 16.6/100,000 [2]. It is the underlying cause in approximately9.2% of children with congenital severe or profound deafness. The incidence of USH in patients with congenital deafness is approximately 3.5–16.6/100,000, which makes this disorder more worthy of our attention, as it accounts for approximately 9.2% of children with congenital severe or profound deafness [3]. Moreover, USH is the most form of syndromic deafness after Pendred syndrome.

The main clinical manifestations of USH include hearing loss, retinitis pigmentosa, and vestibular dysfunction, with different characteristics and onset times [4]. USH is inherited in an autosomal recessive pattern. To date, nine pathogenic genes have been identified and confirmed: *MYO7A*,* USH1C*,* CDH23*,* PCDH15* and *SANS* for Usher type 1(USH1); *USH2A*,* ADGRV1* and *WHRN* for Usher type 2(USH2); and *CLRN1* for Usher type 3(USH3).

Progressive hearing loss is a prominent clinical feature of USH3. Patients are diagnosed with hearing loss at an average age of 8–10 years [5], but show rapid progressive hearing loss between the ages of 20 and 30 years. Retinitis pigmentosa (RP) is usually diagnosed after puberty, at a mean age of 17 years, with night blindness as the first symptom [6, 7]. Unlike the rapid progression of hearing loss, RP progression in patients with USH3 does not appear to differ much from that in patients with USH1 and US2. After puberty, rod degeneration leads to progressive visual field loss, which eventually develops into tubular visual fields and blindness. USH3A patients show different degrees of vestibular dysfunction, and approximately 51% of the tested patients show some abnormalities in vestibular tests [5, 8].

**Fig. 1:**
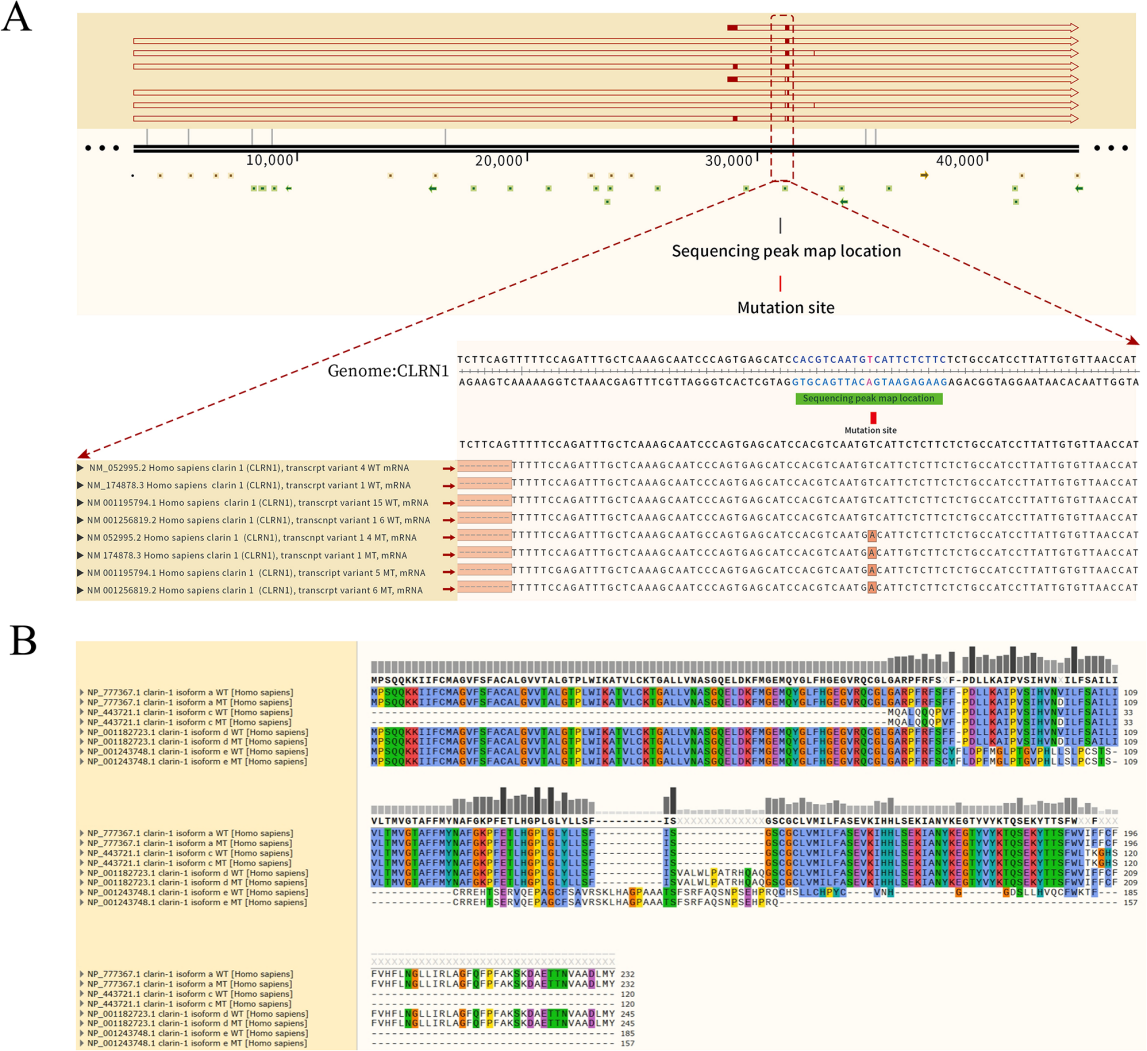
**(A)** The mutation sites were found in different transcripts of the CLRN1. **(B)** Point mutation of CLRN1 can lead to different amino acid sequence changes in different transcripts

As early as 1995, Eeva-Marja Sankila et al. [9] mapped the only gene responsible for USH3 to the locus 3q21-25. Then, in 2002, Avital Adato et al. [10] identified USH3A transcripts in humans and mice. By analyzing USH3A expression patterns, the transcript of this gene was identified in the cochlear hair cells and spiral ganglion cells of mice by in situ hybridization technology, and it was named clarin-1. After nearly a decade of unremitting efforts, the locus of the human *CLRN1* gene was finally determined to be 3q25.1. Similar to other tetraspanins, *CLRN1* retains only a limited hydrophilic region, is subjected to the cytoplasmic or extracellular aqueous phase, and is clearly lacking any functional domain. At least 3 splice isoforms have been reported (Fig. 1). All the known *CLRN1* mutations are located in the 3 exons of isoform a, except an intron mutation in the intron region between exon 0 and exon 0b of isoform e. It was shown that the main isoform of the *CLRN1* gene encodes a 232-amino acid protein predicted to have four transmembrane domains and a glycosylation site in the first extracellular loop [10].

*CLRN1* plays an important role in the morphogenesis and maintenance of hair bundles in mouse cochlear hair cells, and may also have synaptic effects [11, 12]. In vitro biochemical analyses suggested that *CLRN1* functions as a molecular scaffold, recruiting proteins involved in cell adhesion at different plasma membrane regions and playing a role in organizing the actin cytoskeleton organization. Consistent with this function, *Clrn1* knockout (KO) and N48K knockout mice exhibit dysplastic hair bundles at an early age and are severely deaf by postnatal day 21 (P21) [11, 12]. However, similar to other mouse models of USH disease, these mice do not have the ocular phenotype of USH3 patients. Despite the presence of genetic and phenotypic features in clinical patients, the molecular function of *CLRN1* and its relationships and interactions with other Usher gene products have remained difficult to determine.

This chapter describes a molecular etiology study that was conducted for a family with hereditary deafness. There were 2 patients in the family across 3 generations. All patients developed bilateral sensorineural hearing loss (SHL), progressive vision loss and nyctalopia. Through detailed clinical history data, combined with complete physical examinations, imaging examinations and molecular etiology assessments, the molecular pathogenic mechanism of the CLRN1 gene in the immortalized lymphoid cell line was evaluated. This chapter intends to identify the special clinical phenotypes and molecular etiology of families to provide practical and effective genetic counseling for families and descendants and provide more resources for USH3 long-term research in the Chinese population.

## Materials and methods

### Subjects

One three-generation Chinese family with syndromic hearing loss (SHL) was referred to the Department of Otolaryngology and Head and Neck Surgery at Lanzhou University Second Hospital (Lanzhou, China).

### Clinical evaluations

Clinical evaluations, including otoscopic examination, visual reinforcement, audiometry, tympanometry, acoustic reflex, pure-tone audiometry or play audiometry, distortion product evoked otoacoustic emissions (DPOAEs), auditory brainstem responses (ABRs) and auditory steady-state response (ASSR), were completed by an otorhinolaryngologist, ophthalmologist, and a clinical geneticist. Bilateral air conduction (AC) thresholds were determined at octave frequencies of 0.25–8.0 kHz. The AC average thresholds at conversational frequencies of 0.5, 1, 2, and 4 kHz were measured and used to define hearing loss severity. Hearing levels were labeled subtle (16–25 dB), mild (26–40 dB), moderate (41–70 dB), severe (71–95 dB), or profound (95 dB). Best corrected vision, binocular visual evoked potential (VEP), visual field, and fundus examinations were performed on some family members. High-resolution computed tomography (HRCT) was also performed to verify whether family members had complications other than hearing disorders.

### Peripheral blood samples

After informed consent was obtained, blood samples (2 ml + 5 ml) were obtained from all pedigree members, 500 normal control individuals (263 males and 237 females, aged from 18 to 25 years old) and 122 unrelated Chinese families.

### Targeted gene capture and high throughput sequencing

Genomic DNA from peripheral blood leukocytes of the subjects was obtained by the phenol/chloroform method. The DNA was quantified using a Nanodrop 2000 spectrophotometer. Next-generation sequencing (NGS) technology was used to identify the pathogenic genes in this family. During targeted capture and massively parallel sequencing (MPS), the qualified gDNA of the proband (II-4) that met certain criteria was randomly cut using a Covaris S2 focused ultrasound (Covaris, Massachusetts, MA, United States) with an average fragment size of 350–400 bp. The fragment tip was then repaired, connected to an adapter, and analyzed using an Agilent 2100 bioanalyzer. All exon and flanking intron regions, 6 deafness-related mitochondrial regions and 3 miRNAs of 139 deafness-related nuclear genes were captured using GenCap kits (MyGenostics, Beijing, China) (Supplement Tables 1, 2 and 3). The captured sequences were analyzed using the NextSeq 500 Next-generation Sequencer (Illumina Inc., San Diego, CA, USA) for high-throughput sequencing.

**Table 1:** Primer sequences of Sanger sequencing

Primer	primer sequence(5’to3’)
CLRN1-F	CATGGTGGGGACAGCCTTC
CLRN1-R	GCTAGGCCCCAAAACATGAG

### Sanger sequencing

After filtering against multiple databases, all the PCR amplified products were purified with a Millipore plate, and were then sequenced with an ABI 3730 Sequencer (Applied Biosystems, Foster City, CA, USA). The sequence data were analyzed and compared with the reference sequences of the *CLRN1* (NM_001256819.2, ) using the DNAStar 5.0 and BioEdit software packages. The primer sequences are shown in Table 1.

### Cell lines and culture conditions

Immortalized lymphoblastoid cell lines were generated from family members(II-1, II-2 and II-8), and named the cell lines homozygote (Hom), heterozygote (Het), and wild-type (WT), respectively [13]. These cell lines were grown in RPMI 1640 medium with 10% fetal bovine serum (FBS).

### Gene expression analysis

Total cellular RNAs were extracted from various cells using TRIzol reagent (Invitrogen, 15596026) and reverse transcribed into cDNA using PrimeScript II 1st Strand cDNA Synthesis Kit (Takara, 6210 A). qPCR was performed on the Applied Biosystems 7900HT Fast Real-Time PCR System. Data were analyzed using the 7900 System SDS RQ Manager Software, and relative gene expression was determined using the 2–ΔΔCt method using GAPDH as a housekeeping gene. Primer sequences for this study are listed in Table 2.

**Table 2:** *CLRN1*: c.474T > A (p.Cys158Ter) pathogenic analysis

Tools	Pathogenicity	Score
PROVEAN	Deleterious	-5.08
SIFT	Damaging	0.001
PolyPhen	Probably damaging	0.925
Mutation Taster	Disease causing	1
MutationAssessor	stop gain	

**Table 3:** Primer sequence of RT - qPCR

Primer	primer sequence(5’to3’)
CLRN1-F	TGAGGCATTGACGAGCAGAG
CLRN1-R	TCGGAGTTGTGACAGCCTTG
GAPDH-F	GGAGTCCACTGGCGTCTTCA
GAPDH-R	GTCATGAGTCCTTCCACGATACC

According to the different transcripts of CLRN1, specific primers and probes were designed in NCBI (Table 3).With Immortalized lymphoblastoid cell lines RNAs for testing template, with 2 x T5 fast QPCR mix (probe) is used to detect the computer, amplification system components are as follows:

**Table:** 

Component	Volume
2*One Step Buffer	10 ul
Enzyme Mix (UDG plus)	1 ul
10 µM Primer F	1 ul
10 µM Primer R	1 ul
Probe (10µM)	1 ul
Template (RNA)	5 ul
50×ROX Reference Dye I	0.4
ddH2O	0.6 ul
Total	20 ul

The above amplification system amplification detection according to the following expansion program:

**Table:** 

Temperature (℃)	StageType	Keep Temperature Time (s)	Temperature Rate (℃/s)	Cycles
50	StageHold	900	2	1
95		180	2	
94	StageCycling	15	2	45
60		30	2	

### In silico analysis

The Alphafold2 prediction software (https://www.alphafold.ebi.ac.uk/) was used to predict the possible influence of this mutation in the different transcripts.

### Plasmid construction and cell transduction

“CLRN1- wild-type or c.474T > A Mutation binding site (*CLRN1*, NM_001256819.2)” and “CLRN1- wild-type or c.302T > A Mutation binding site ( *CLRN1*, NM_174878.3)” expression plasmids were derived from pRP[Exp]-EGFP/Puro-EF1A > FLAG. HEK293T Cell lines were cultured as adherent monolayers in plastic flasks (Beyotime) in DMEM (Hyclone) supplemented with 10% foetal bovine serum (Gemini) and 1× Antibiotic-Antimycotic (Gibco). Cells were cultured in 5% CO2/95% humidified air at 37 °C. Cell lines were transfected at 70–80% confluence in 6-well plates (Beyotime) using Lipofectamine™ 2000 transfection reagent (Thermo Fisher Scientific) as recommended by the manufacturer (4 µL Lipofectamine™ 2000 and 1.6 µg plasmid were each added to 100 µL Opti-MEM and combined per transfection). 1.5 × 105 cells were plated per well in 1 mL DMEM plus 10% FBS 24 h prior to transfection. Cell lines in 6-well tissue culture plates in 3 mL of medium were transduced when 70–80% confluent with 1 mL of unconcentrated lentivirus followed by incubation at 37 °C. When the coverage of HEK293T cells reached 90 to 95%, the cells were harvested and total protein was extracted.

### Western blot assays

Western blot analysis was performed using 25 µg of total cellular proteins isolated from human cell lines, as detailed elsewhere [14]. The primary antibodies obtained from different companies were as follows: Invitrogen (anti-CLRN1 [PA5-70444] ) and beta Tubulin Rabbit Monoclonal Antibody [Beyotime; AF1216 ]), and goat anti–rabbit IgG (Jackson ImmunoResearch Laboratories, West Grove, 111–005144) were used as a secondary antibody. Protein signals were visualized using the ECL system (CWBIO).

## Results

### Clinical phenotype analysis

**Fig. 2:**
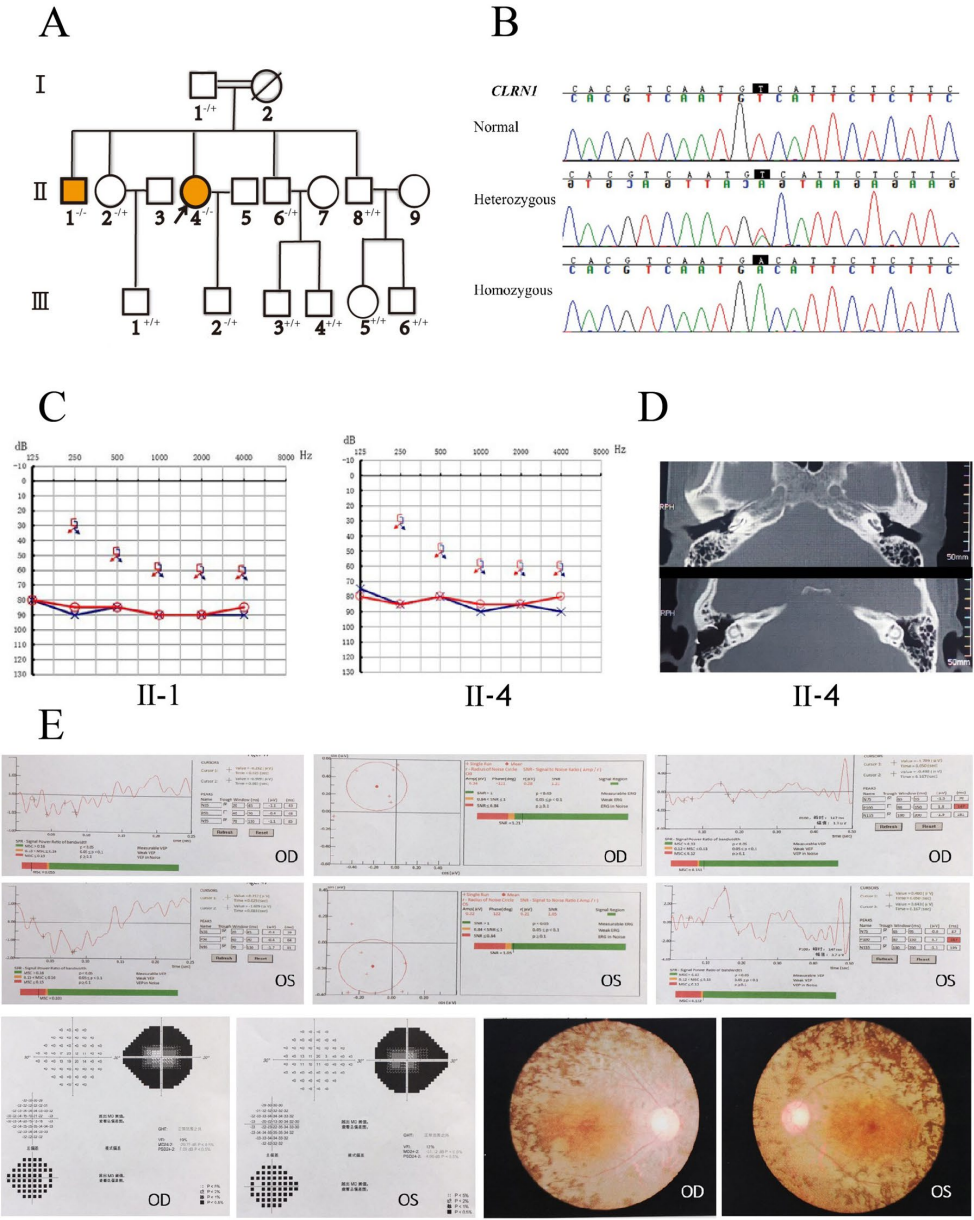
Pedigree diagrams **(A)**, Mutation information **(B)**, audiograms information **(C)**, the temporal CT scan **(D)**, and Ophthalmology information **(E)**.**(A)**, Pedigree diagram of the family. Filled black symbols for males (squares) and females (circles) represent affected individuals (II-1,II-4), and empty represent unaffected ones. An arrow denotes the proband. (**(B)**), DNA sequence chromatograms shows a novel homozygous variant CLRN1 c.474T > A (P.Cys158Ter, NM_001256819.2) or c.302T > A (p.Val101Asp, NM_174878.3) of II-1,II-4.**(C)**, Audiograms of the affected siblings. They exhibited bilateral severe sensorineural deafness. The horizontal axis shows tone frequency (Hz); the vertical axis shows hearing level (dB). **(D)**, The temporal CT scan of siblings. The results of the temporal CT scan showed no obvious abnormality. **(E)**Binocular VEP examination showed that the P100 peak latent time delay and amplitude were decreased in both eyes of the proband. Binocular visual field examination results suggested a binocular tubular field. The color fundus of both eyes showed that the boundary of the optic disc of both eyes was clear and waxy yellow, the reflection of the macular area was not clear, the retinal blood vessels were narrowed, the retina was grayish, and the equatorial retina was pigmented

Two patients in a family with inherited deafness across 3 generations were included. As shown in Fig. 2A, this family exhibited autosomal recessive inheritance of hearing loss. Proband II-4 was a 47-year-old female whose parents I-1 and I-2 were consanguineous. She had postlingual profound SHL in both ears (Fig. 2C) and poor visual acuity in both eyes. At approximately 10 years of age, she developed binaural progressive hearing loss; at the age of 13, she gradually developed visual impairment with nyctalopia. In the past 10 years, her binocular vision has decreased significantly. Examination in the Ophthalmology Department (as shown in Fig. 2E) revealed that her best corrected visual acuity (BCVA) was 0.4 and 0.7 for the left and right eyes, respectively. Binocular VEP examination showed that the P100 peak latent time delay and amplitude were decreased in both eyes. Binocular visual field examination results suggested a binocular tubular field. The color fundus of both eyes showed that the boundary of the optic disc of both eyes was clear and waxy yellow, the reflection of the macular area was not clear, the retinal blood vessels were narrowed, the retina was grayish, and the equatorial retina was pigmented; these characteristics are a typical manifestation of advanced RP. The patient had no obvious symptoms of abnormal vestibular function, such as walking deviation and instability. CT examination of the temporal bone showed that the bilateral external auditory meatus, middle ear and inner ear had no obvious developmental abnormalities (Fig. 2D). In 2015, the patient had a cochlear implant (NUROTRON) in the right ear, and postoperative recovery went well.

II-1 was a patient with congenital deafness, poor hearing response after birth, poor vision since childhood though no eye examination was performed. He had lost his eyesight and the ability to take care of himself.

### Targeted high-throughput sequencing and verification results of coseparation

**Fig. 3:**
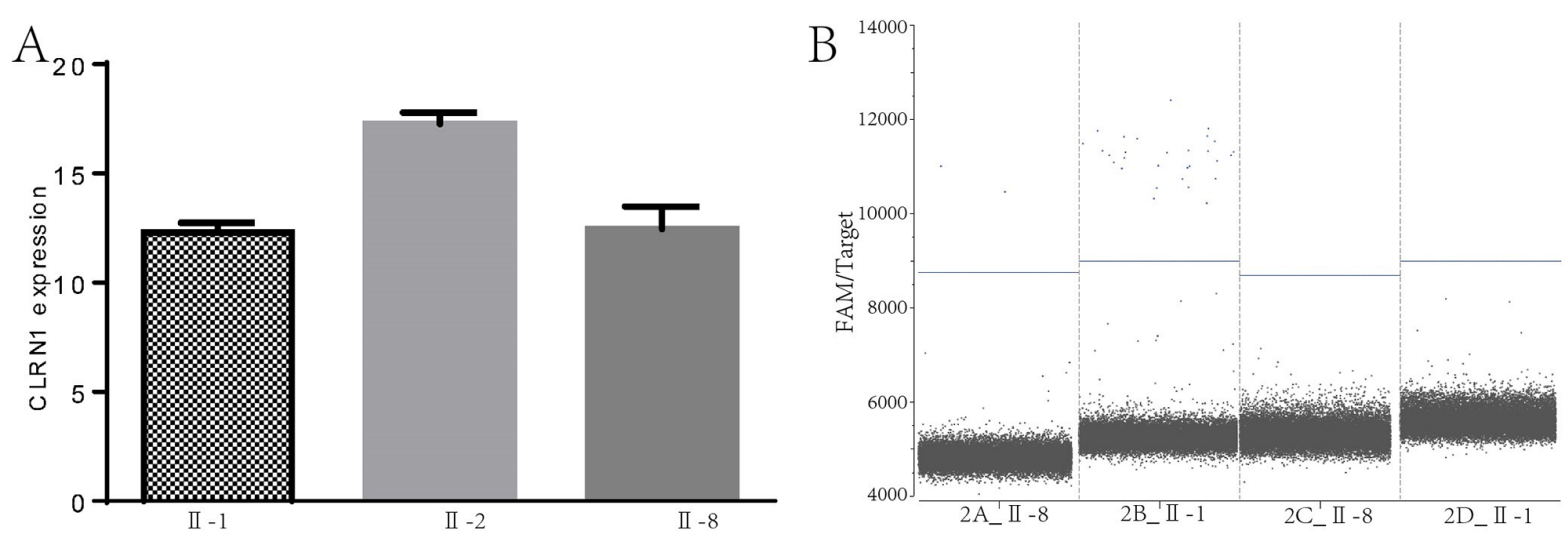
**(A)**, Primers were designed in the common exon region of all transcripts of the *CLRN1*, and the PCR results indicated that homozygous mutation did not affect the stability of its mRNA and did not alter the transcription of *CLRN1. ***(B)**, specific primers were set for NM_174878.3 and NM_001256819.2, Digital PCR results indicated that the non-canonical transcript(NM_001256819.2) was not expressed in these ILCL. The variant is c.302T > A does not lead to reduced transcription of canonical transcript (NM_174878.3), even an increase in the amount of transcription

We sequenced all the coding exons plus ~ 100 bp of the flanking intronic sequences of 139 deafness genes in the proband (II-4). The average depth of cover for targeted regions among 110–160×, with > 98% having > 20×depth of coverage. Our results from panel analysis thus largely excluded not only mutations in the coding sequences of Usher syndrome genes and genes causing similar syndromes, such as ABHD12 mutations and *HARS* mutations, but also simultaneous mutations in genes associated with deafness and an RP leading to a phenotype mimicking Usher syndrome. Finally, one novel homozygous variant at transcript NM_001256819.2 in which pathogenicity has not always been reported, CLRN1:c.474T > A (P.Cys158Ter), was detected in both patients II-4 and II-1. If the variant is c.302T > A, this transcript is p.Val101Asp (shown at Figs. 2B and 3), based on the MANE select transcript NM_174878.3.

### Sanger sequencing

Sanger sequencing of normal-hearing family members revealed that I-1, II-2, II-6, and III-2 all carried the monozygotic mutation *CLRN1* c.474T > A (P.Cys158Ter, NM_001256819.2) or c.302T > A (p.Val101Asp, NM_174878.3), and the variant was not detected in other family members. Furthermore, the variant has not yet been identified in a large population database and has not been reported in the literature .

We screened 500 normal Chinese individuals and 122 unrelated Chinese families with apparent hearing loss but did not detect the loci of *CLRN1*: c.474T > A (or c.302T > A) by Sanger sequencing.

### RT‒PCR analysis of CLRN1 expression in an immortalized lymphocyte line and HEK293T cells

We constructed a family-derived immortalized lymphoid cell line (ILCL) by collecting peripheral blood from family members (II-1, II-2 and II-8) to establish the Hom, Het, and WT cell lines, respectively.

**Fig. 4:**
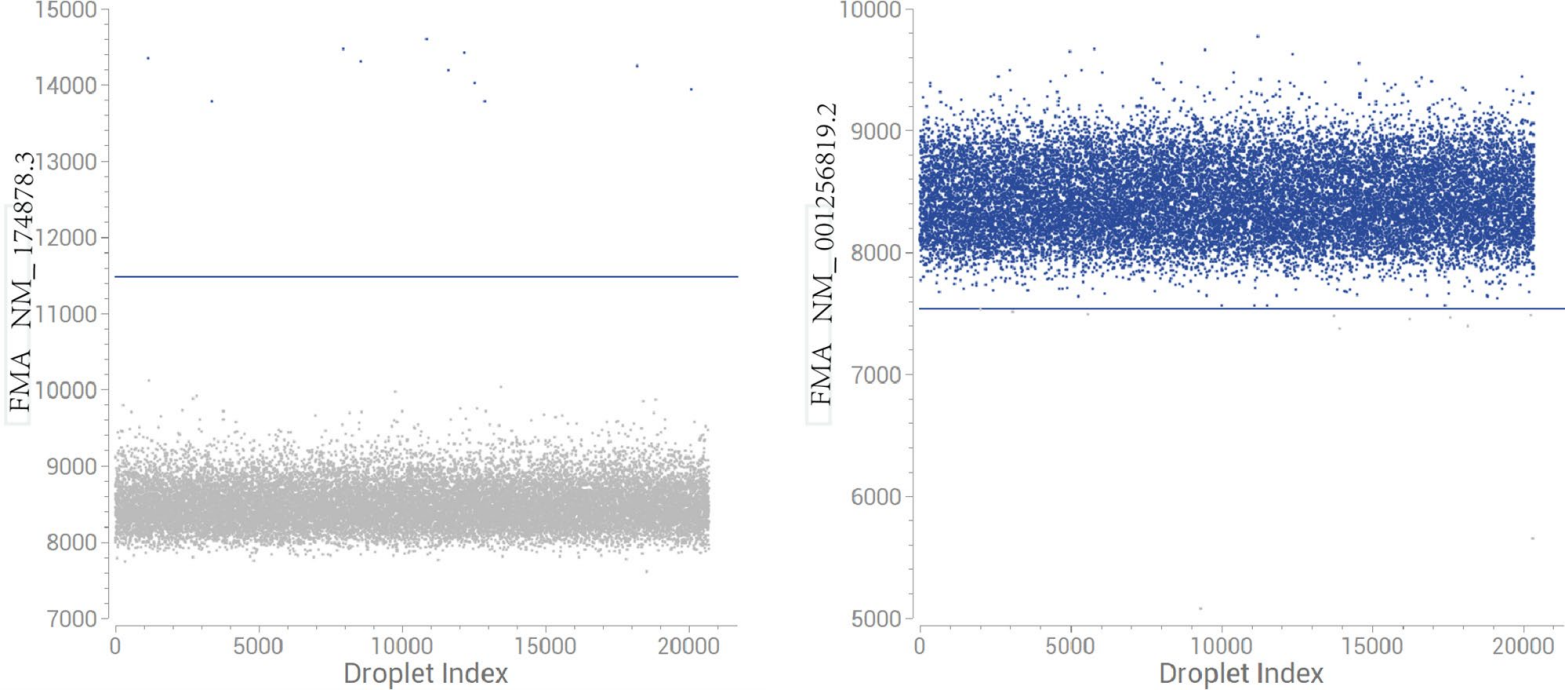
The transcript NM_001256819.2 was highly expressed in HEK293T

To further evaluate the effect of c.474T > A (or c.302T > A) on ILCL differentiation, we performed qPCR analysis of CLRN1 using total RNA isolated from Hom, Het, and WT as templates. The results revealed that the homozygous mutation c.474T > A (or c.302T > A) did not affect the stability of its mRNA and did not alter the transcription of *CLRN1* (Fig. 4A).

**Table 4:** Primer and probe sequence of Digital qPCR

Primer and probe	sequence
NM-001256819.2-F	GGCTTTTCCACGGAGAGGGT
NM-001256819.2-R	GGGAGTCCCATGAAGGGGTC
NM-001256819.2-P	TCGGTTCTCATGCTATT
NM-174878.3-F	AGTGTGGGTTGGGAGCAAGG
NM-174878.3-R	CAGCCACAGGAGCCTGAAATG
NM-174878.3-P	TGGAAAAAATGAGAACC

In order to verify the expression of NM_001256819.2 and NM_174878.3 in ILCLs, specific primers were defined for these two transcripts, and digital PCR was performed. The results indicated that the noncanonical transcript (NM_001256819.2) was not expressed in these ILCLs. Moreover, the results suggest that the variant c.302T > A does not lead to reduced transcription of the canonical transcript (NM_174878.3) and may even increase the amount of transcription (Table 4; Fig. 3B).

**Table 5:** The results of Digital PCR detection of different transcripts of clrn1 gene in the patient’s immortalized lymphocyte line

Transcript	Well Name	Nb Droples	C(cp/ul)	Reaction system(ul)	Amount of RNA detected(ul)	The copy number(copies/ul)
NM-174878.3	1. 2A_II-8	19,213	0.13	20	5	0.52
	2. 2B_II-1	19,828	1.83	20	5	7.32
NM-001256819.2	3. 2C_II-8	18,948	0	20	5	0
	4. 2D_II-1	19,572	0	20	5	0

**Table 6:** The results of Digital PCR detection of different transcripts of clrn1 gene in the HEK293T

Transcript	Nb Droples	C(cp/ul)	Reaction system(ul)	Amount of RNA detected(ul)	The copy number(copies/ul)	
NM-174878.3	20,717	0.66	20	5	6.6	
NM-001256819.2	20,351	9403.74	20	5	94037.4	

To determine whether NM_001256819.2 exists in human cells, we selected HEK293T cells, extracted total RNA and performed digital PCR. The results suggested that NM_001256819.2 was highly expressed in HEK293T cells (Table 5).

### The p.Cys158Ter mutation affected the Clarin-1 function

**Fig. 5:**
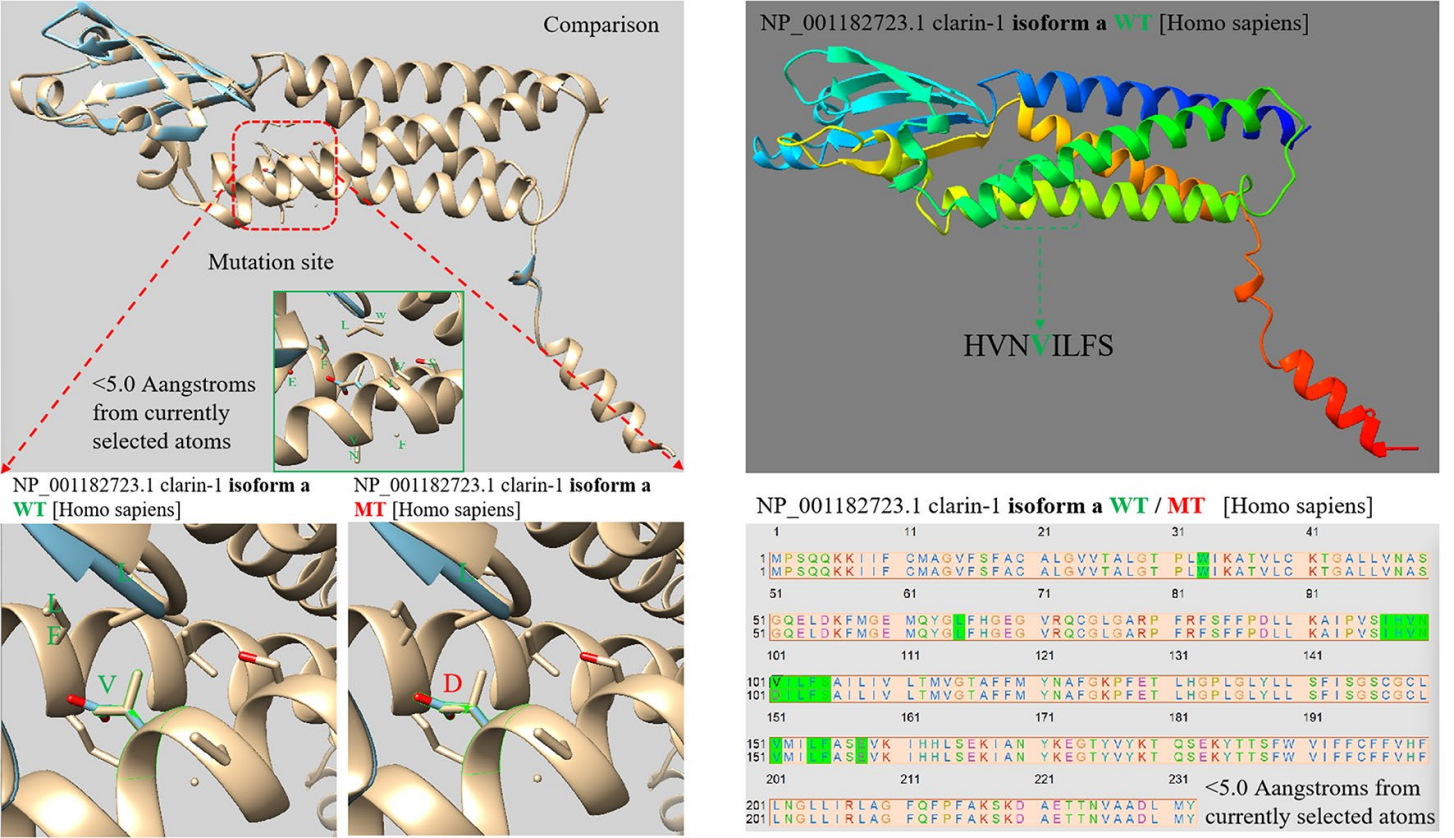
The Alphafold2 prediction software showed that CLRN1c.302T > A caused an alpha folding abnormal and did not cause other spatial structure changes

**Fig. 6:**
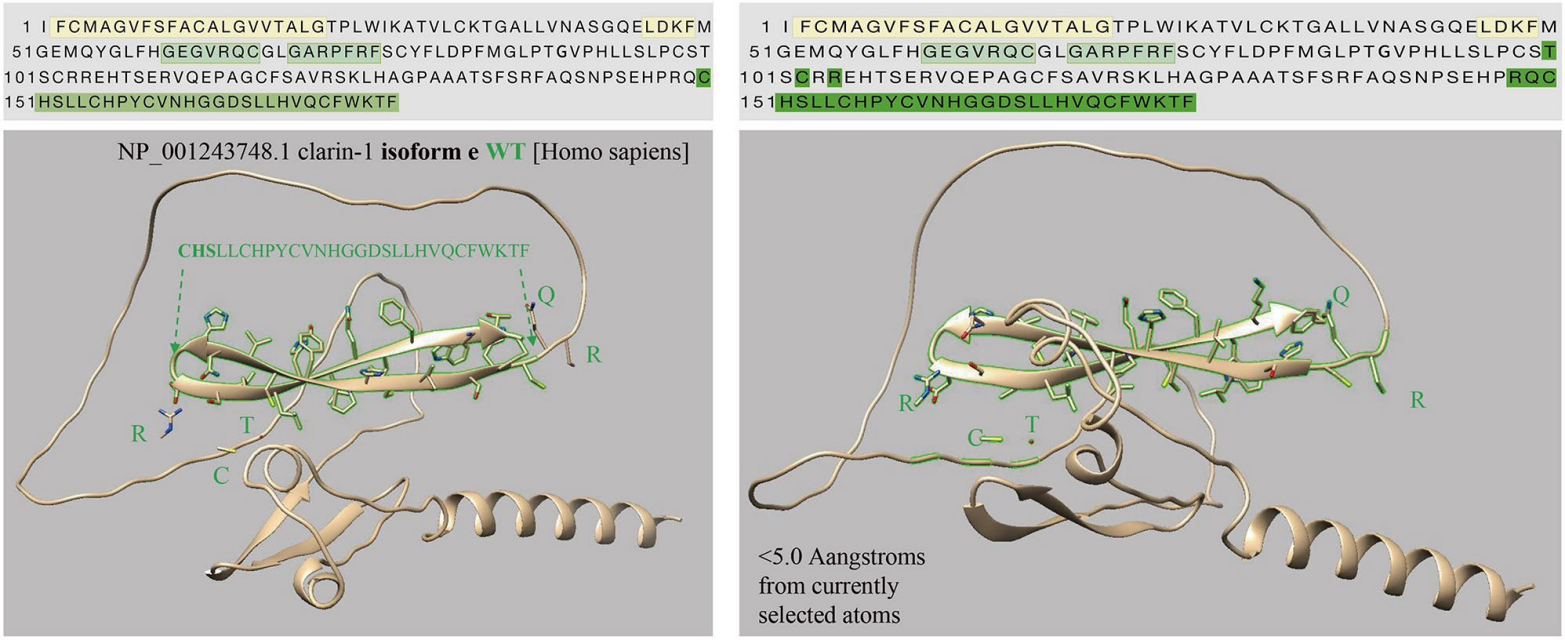
The *CLRN1*c.474T > A(NM_001256819.2)mutation occurs at the 158th amino acid, and the corresponding cDNA produces a stop codon, resulting in early termination of translation

The *CLRN1*c.302T > A (NM_174878.3) mutation causes the 101 valine residue to become aspartate. As shown in Fig. 5, the Alphafold2 prediction software revealed that *CLRN1*c.302T > A caused an abnormal alpha folding and did not cause other spatial structure changes. However, the *CLRN1*c.474T > A (NM_001256819.2) mutation occurs at the 158th amino acid, and the corresponding cDNA produces a stop codon, resulting in the early termination of translation. The loss of large amino acid fragments ultimately affects the protein spatial structure (Fig. 6). The predictive results of the pathogenicity of *CLRN1*c.474T > A (NM_001256819.2) are shown in (Tables 6, 7).

**Table 7:** *CLRN1*: c.474T > A (p.Cys158Ter) pathogenic analysis

Tools	Pathogenicity	Score
PROVEAN	Deleterious	-5.08
SIFT	Damaging	0.001
PolyPhen	Probably damaging	0.925
Mutation Taster	Disease causing	1
MutationAssessor	stop gain	

**Fig. 7:**
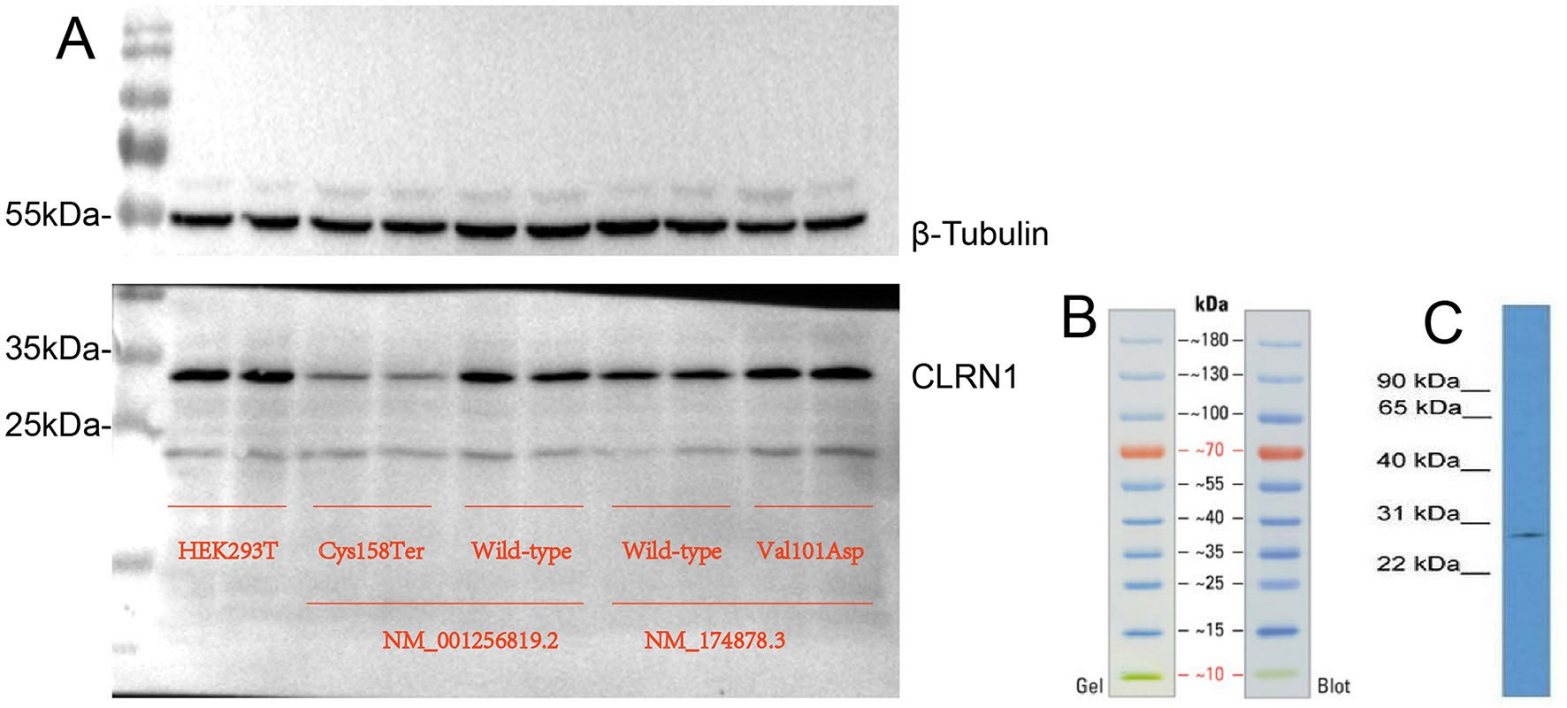
(**A**) Over-expression of 474T > A (p.Cys158Ter, NM_001256819.2) caused the loss of Clrn1 protein, but the c.302T > A(Val101Asp, NM_174878.3) did not. (**B**) The marker (**C**) CLRN1 Antibody (PA5-70444) in WB. Western blot analysis of CLRN1 on human brain cells. The sample was probed with a CLRN1 polyclonal antibody (Product # PA5-70444) using a primary antibody dilution of 0.2–1.0 µg/mL

To experimentally test the predicted effect of the mutation on *CLRN1*, we used pRP[Exp]-EGFP/Puro-EF1A > FLAG as a carrier, constructed “CLRN1- wild-type or c.474T > A mutation binding site (*CLRN1*, NM_001256819.2)” and “CLRN1- wild-type or c.302T > A mutation binding site (*CLRN1*, NM_174878.3)” expression plasmids, and transfected them into HEK293T cells. As shown in Fig. 7A, the proteins were extracted and detected via WB. The results revealed that the overexpression of 474T > A (p.Cys158Ter, NM_001256819.2) caused the loss of the Clrn1 protein, whereas c.302T > A (Val101Asp, NM_174878.3) did not.

## Discussion

In this study, we investigated the clinical phenotype and molecular etiology of 2 persons in a family with inherited deafness across 3 generations. According to the progressive hearing loss and visual acuity loss in the proband and in some patients in the family, accompanied by the proband’s ophthalmic examination, USH3 was initially diagnosed. Two patients carried a homozygous variation of *CLRN1* c.474T > A (P.Cys158Ter, NM_001256819.2) or c.302T > A (p.Val101Asp, NM_174878.3). Sanger sequencing was subsequently used to verify the likely pathogenic variants sites in the above two branches, and the verification structure revealed that the homozygous variation of *CLRN1* c.474T > A (P.Cys158Ter, NM_001256819.2) or c.302T > A (p.Val101Asp, NM_174878.3) was coisolated with the genotype‒phenotype of the family.

USH3 mainly manifests as postlinguistic progressive bilateral symmetrical SHL, and vestibular function may decline, with or without RP occurring before 20 years of age [7]. However, patients with USH3 showed significant genetic heterogeneity and phenotypic specificity. At present, a *CLRN1* gene mutation is believed to cause USH3A, and an *HARS* gene mutation causes USH 3B [15]. USH3 is relatively rare compared to USH1 and USH2, although Hope et al. [16] reported that USH3 accounted for approximately 20% of USH in Birmingham. Ness et al. [5]reported that this subtype accounted for approximately 40% of all USH patients in Finnish and Ashkenazi Jews. However, in the Spanish population, a study of 89 USH patients with no instances of USH3 was followed by analysis of a sample of 131 families with only 8 USH3 cases, an incidence of only 6% [8]. Familial USH3 has not been reported in the Chinese population; only sporadic cases have been reported due to eye disease.

Due to the high phenotypic heterogeneity of USH3 patients not only among different families but also within the same family, Ness et al. [5] believed that the phenotype of USH3 patients with SHL was relatively consistent, and this clinical phenotype was used as an important distinguishing feature to distinguish USH3 from UHS2 and USH1. Another clinical manifestation commonly used to distinguish USH3 from UHS2 and USH1 is the onset time of deafness.

Postlingual SHL is characteristic of USH3, whereas prelingual hearing loss is a feature of USH. In this study, the proband had typical USH3 clinical symptoms—from hearing, vision and typical RP—the onset age and development were similar to those found in the literature, but her older brother (II-1) developed binaural severe SHL after birth, night blindness since childhood and, poor binocular vision; moreover, his binocular vision is almost completely gone. Therefore, the clinical manifestations of USH3 patients in this family were not identical. In 1999, Adato et al. [17] found 2 patients with different clinical phenotypes in a nonclose family of Jewish Yemenis, including 1 patient with the USH1 clinical phenotype and the other with the USH3 clinical phenotype. In subsequent studies, the authors reported that the different clinical phenotypes in the same family occurred were because patients with the USH1 phenotype carried the complex heterozygous variation in MYO7A, while patients with the homozygous variation of the USH3 haplotype presented the USH3 phenotype. Ness et al. [5] also found that among 40 Ashkenazi Jews with USH, 16 patients were clinically classified as USH3. Although all patients had *CLRN1* mutations, those with homozygous p.N48K showed more severe clinical phenotypes, with particularly prominent audiological manifestations, and the onset age ranged from infancy to over 35 years old. Aller et al. [8] reported that three Spanish families with USH also presented with postlingual bilateral symmetric severe SHL (at age 6: average hearing threshold 70 dB; age 17: average hearing threshold > 90 dB).In a study of *CLRN1*^p.Y176X^ knockout mice, the clinical phenotype of the knockout mice was very similar to that of patients with the *CLRN1*^p.Y176X^ mutation; however, hearing loss occurred early and progressed rapidly, and vestibular function loss occurred much later than hearing loss. These results indicate that clinical confusion may exist between USH3 and USH1 or between USH3 and USH2. However, due to delayed and progressive hearing loss, some patients whose hearing is clinically consistent with that of USH3 have been found to carry variants of *USH2A*,* USH1B* and *USH1D*. Therefore, according to our results and previously reported findings, progressive SHL and postlingual deafness are not important criteria for the diagnosis and differentiation of USH3. With the gradual increase in case data, USH3 clinical phenotypes have appeared in more forms than clinical phenotypes for USH diagnosis, and the genetic diagnosis has become much clearer and more reliable.

The main significance of this study is embodied in the following aspects. The NCBI website (*CLRN1* clarin 1 [[*Homo sapiens*(human)] -Gene-NCBI (nih.gov)) recorded four transcripts (Fig. 1), which transcribed different amino acid sequences due to different exons contained. This partly explains why CLRN1 mutations cause heterogeneity in the clinical phenotype of patients. NM_174878.3 and NP_777367(isoform a is encoded by transcript variant 1) contain exons 0-1-2, encoding a clarin-1 protein containing 232 amino acids of approximately 25.8 kDa [18]. All the known *CLRN1* mutations are located in the 3 exons of isoform a, except an intron variant in the intron region between exon 0 and exon 0b of isoform e. With the exception of the p.Y176X and P.P.120 K mutations found in Finnish populations [19] and the p.N48K variant found in Ashkenazi Jews [20], most of the other variants have been found in single families. In addition to these three common mutations, 21 missense and nonsense mutations, 2 occurrences of abnormal splicing, 7 deletion mutations, and 3 insertion mutations have been reported to date (Human Gene Mutation Database, Deadline 2023.03).

In 2017, Khan et al. [21] reported a *CLRN1* intron mutation c.254–649T > G(NM_001256819.1, isoform e)in an inbred family from the Arabian Peninsula diagnosed with USH1, located in the intron region between exon 0 and exon 0b. Through minigene splicing experiments, it was proven that the mutation caused abnormal splicing of exons, resulting in frameshift and early appearance of stop codons. The authors then tested for the mutation site in seven untested Saudi USH1 patients with a related genetic mutation and found that two of them carried the mutation. Pertinently, c.254–649T > G, which is also the human pathogenic mutation reported to date for non-NM_174878.3 transcripts, represents a founder allele that may significantly contribute to deaf-blindness in people on the Arabian Peninsula.

Using the prediction software Alphafold2, we found that the homozygous variants [*CLRN1* c.474T > A (P.Cys158Ter, NM_001256819.2) or c.302T > A (p.Val101Asp, NM_174878.3)] carried by the familial patients had different effects on different transcripts of *CLRN1*. Although we did not obtain direct evidence from patient-derived ILCLs, we confirmed the presence of specific transcripts in HEK293T cells, and through plasmid expression experiments, we observed protein expression differences caused by c.474T > A (P.Cys158Ter, NM_001256819.2). The mechanism may be that the mutation of noncanonical NM_001256819.2 leads to alternative splicing of the canonical transcript, which eventually leads to protein degradation.

The *CLRN1*c.474T > A (p.Cys158Ter) variant has not been reported before. To the best of the authors’ knowledge, there is no report about this variant in any databases, including The Single Nucleotide Polymorphism Database, The Human Gene Mutation Database, 1000 Genomes Project and ClinVar and Exome Sequencing Project v. 6500. As suggested by the ACMG/AMP guidelines, a pathogenicity analysis was performed: (a) According to the recommendations of ACMG/AMP guidelines, pathogenicity analysis was performed: (a) Through in vitro experiment verification of lymphocyte line construction and plasmid transfection experiment, it was found that *CLRN1*c.474T > A (p.Cys158Ter) variant led to abnormal protein expression and impaired gene function (strong pathogenic evidence PS3); (b) the novel variant *CLRN1*c.474T > A (p.Cys158Ter) was not identified in control groups, the frequency in the normal population database is “-” when compared that of GnomAD (moderate pathogenic evidence PM2); (c) Hearing loss and RP expression in proband and older siblings are highly characteristic of the *CLRN1* gene (supporting evidence for pathogenesis, PP4) and (e) USH3 is a recessive genetic disorder. The affected siblings were found to carry *CLRN1*c.474T > A (p.Cys158Ter) homozygous mutations, while the remaining family members had either heterozygous or wild type mutations, confirming that the phenotype of hearing loss and retinitis pigmentosa co-segregated with genotype in this family (supportive pathogenic evidence, PP1). Taken together, the evidence for the c.474T > A mutation is “PS3 + PM2 + PP4 + PP1” and is judged to be a pathogenic mutation (very strong pathogenic evidence). The *CLRN1*c.474T > A (p.Cys158Ter, NM_001256819.2) mutation underlies the first report of USH3 in the Chinese population.

## Conclusion

In summary, we determined that a homozygous variant of *CLRN1*c.474T > A (p.Cys158Ter, NM_001256819.2) caused USH3 in a Chinese family because the transcript of this mutation was not the version commonly found in Finnish and Ashkenazi Jewish populations. Therefore, the discovery of this mutation has certain reference value for the study of the pathogenesis and prevention of USH3A, and particular attention should be paid to different transcripts.

## Data Availability

All data generated or analysed during this study are included in this published article.
